# Postural effects on cerebral blood flow and autoregulation

**DOI:** 10.14814/phy2.13150

**Published:** 2017-02-27

**Authors:** Zachary K. Garrett, James Pearson, Andrew W. Subudhi

**Affiliations:** ^1^ Department of BiologyUniversity of Colorado Colorado SpringsColorado SpringsColorado

**Keywords:** Cardiac output, cerebrovascular, end‐tidal carbon dioxide, hydrostatic pressure, seated, supine

## Abstract

Cerebral autoregulation (CA) is thought to maintain relatively constant cerebral blood flow (CBF) across normal blood pressures. To determine if postural changes alter CA, we measured cerebral blood flow velocity (CBFv) in the middle cerebral arteries, mean arterial blood pressure (MABP), cardiac output (Q), and end‐tidal carbon dioxide (PETCO_2_) in 18 healthy individuals (11 female and seven male; 26 ± 9 years) during repeated periods of supine and seated rest. Multiple regression was used to evaluate the influence of PETCO_2_, MABP, Q, and hydrostatic pressure on CBFv. Static CA was assessed by evaluating absolute changes in steady‐state CBFv. Dynamic CA was assessed by transfer function analysis of the CBFv response to spontaneous oscillations in MABP. In the seated versus supine posture, MABP (67.2 ± 7.2 vs. 84.2 ± 12.1 mmHg; *P* < 0.001), CBFv (55.2 ± 9.1 vs. 63.6 ± 10.6 cm/sec; *P* < 0.001) and PETCO_2_ (29.1 ± 2.6 vs. 30.9 ± 2.3 mmHg; *P* < 0.001) were reduced. Changes in CBFv were not explained by variance in PETCO_2_, MABP, Q, or hydrostatic pressure. A reduction in MABP to CBFv transfer function gain while seated (*P* < 0.01) was explained by changes in the power spectrum of MABP, not CBFv. Our findings suggest that changes in steady‐state cerebral hemodynamics between postures do not appear to have a large functional consequence on the dynamic regulation of CBF.

## Introduction

The human brain comprises only a small fraction (<5%) of the body's total mass yet consumes 20% of the body's total oxygen uptake (Lassen 1959). The need to maintain oxygen delivery makes the human brain particularly sensitive to changes in blood flow. As such, the cerebral vasculature must be capable of adjusting blood flow throughout a wide range of physiological states and challenges (Willie et al. 2014). Despite decades of research investigating mechanisms governing cerebral blood flow (CBF), precisely how the body regulates flow amidst postural changes remains unresolved.

The term cerebral autoregulation (CA) encompasses the integrated myogenic, metabolic, and neurologic adjustments made to maintain steady CBF, and is subclassified into static and dynamic components (Paulson et al. 1990). Static CA refers to the relationship between mean arterial blood pressure (MABP) and CBF while MABP is in a steady state, spanning several minutes to hours. Dynamic CA refers to the compensatory response in CBF to a change in MABP, typically over a duration of a few seconds.

Recent research has challenged the classical definition of static autoregulation, that CBF remains constant when MABP is within the range of 60–150 mmHg (Lassen 1959), with some suggesting this range is either much narrower [85 to 115 mmHg (Långsjö et al. 2005; Ogoh et al. 2008; Chan et al. 2011)] or even absent (Lucas et al. 2010). A more passive pressure–flow relationship across normal MABP (Lucas et al. 2010) might help explain why steady‐state CBF is reduced when one moves from a supine to upright posture, yet it is unclear how this construct would affect our understanding of dynamic CA between postures.

Although some studies have reported differences in dynamic CA between postures (Deegan et al. 2010; Sato et al. 2012), others have not (Romero et al. 2011; Gelinas et al. 2012). Differences in methods used to provoke changes in MABP and analysis techniques used to quantify the cerebral pressure–flow relationship, make it difficult to compare results and reach a consensus on the effects of posture on dynamic CA. As a means to improve standardization in the field, the cerebral autoregulation network (CARNet) group recently published a set of methodological guidelines and algorithms for the analysis of spontaneous fluctuations in resting MABP and CBF by transfer function analysis (TFA) (Claassen et al. 2016). In this study, we sought to compare indices of static and dynamic CA in supine and seated positions to investigate the influence of posture on cerebral pressure–flow relationships. We hypothesized that a passive MABP‐CBF relation would help explain imperfect static CA and that TFA analysis, using the CARNet guidelines would reveal no functional difference in dynamic CA between postures.

## Methods

### Subjects

Approval from the University of Colorado, Colorado Springs (UCCS) was obtained from the Institutional Review Board (#15–088) in accordance with the Declaration of Helsinki. Eighteen participants (seven male, 11 female) volunteered for the study (Table 1). All participants were healthy and not taking any prescription medications. Participants were told to abstain from caffeine, alcohol, and strenuous exercise for 12 h, and dietary supplements for 72 h, prior to testing. All participants had resided in Colorado Springs for 6 months or more, and were considered acclimated to low altitude (1949 m).

**Table 1:** Subject demographics

	Weight (kg)	Height (cm)	Age (years)
Male (*n* = 7)	82.2 ± 16.0	181 ± 6	30 ± 13
Female (*n* = 11)	62.7 ± 6.5	166 ± 5	23 ± 4
Total (*n* = 18)	70.3 ± 14.6	172 ± 9	26 ± 9

Mean ± SD.

### Instrumentation

Participants were instrumented in a seated position on a modified reclining chair (Ostrich Deluxe Chair, Deltess Corp., Deptford, NJ) mounted on a custom built wooden platform. The platform and modifications allowed a seatback angle of 90° when seated and 0° when supine. Cerebral blood flow velocity (CBFv) in both middle cerebral arteries (MCAs) was insonated through the temporal windows using a 2‐MHz transcranial Doppler (TCD) probe (Spencer Technologies, Seattle, WA) secured to a headset (Mark 600, Spencer Technologies, Seattle, WA). The MCAs were insonated at depths of 45–50 mm. A nasal cannula was placed inside one nostril to sample expired gases and determine partial pressure of end‐tidal carbon dioxide (PETCO_2_; Oxigraf O2cap gas analyzer, Mountain View, CA). Photoplethysmography (Nexfin HD, Irvine, CA) and pulse contour methods were used to obtain beat‐by‐beat MABP and cardiac output (Q) from a finger cuff placed over the 2nd phalange of the right index or middle finger (Jellema et al. 1999; Eeftinck Schattenkerk et al. 2009). MABP measurements were corrected for hydrostatic pressure based on the vertical distance from the measurement site to the level of the heart (MABP_heart_). An automated sphygmomanometer (Mobil‐o‐graph, I.E.M. GmbH, Stolberg, Germany) was placed over the brachial artery (heart level) contralateral to the finger cuff sensor to verify photoplethysmograph measures of MABP. Continuous electrocardiograph (ECG) signals were recorded, using a standard lead II electrode configuration. All sensor signals were continuously monitored throughout the protocol to ensure signal strength. Analog signals were captured in digital format (200 Hz) via a data acquisition system (Powerlab 16SP, ADInstruments, Colorado Springs, CO) and stored for analysis.

### Protocol

Participants were instrumented in the seated posture with their feet flat on the floor and their torsos and heads supported by the chair. In the supine posture, the chair also supported each participant's head, torso, and legs. The starting posture was randomized and balanced within participants. The protocol began with 5 min of quiet, eyes‐open rest to ensure steady‐state hemodynamics. Following the initial rest period, 5 min of data were collected. Participants remained in the chair as the seatback and footrest were adjusted to passively move them to the next posture (either supine to seated or seated to supine). After a 5‐min equilibration period, 5 min of data were collected. This sequence was repeated to obtain two sets of measurements in each posture.

### Data analysis

From the continuous measurements, cardiac cycles and breaths were identified to calculate MABP and PETCO_2_ in mmHg, and mean CBFv in cm/s (LabChart v7, ADInstruments, Colorado Springs, CO). For each individual, the last 30–60s of data from both trials in each position were used to calculate a single average value for the supine trials and a single average value for the seated values.

Static CA was assessed by comparing the absolute CBFv between supine and seated postures and considered perfect if CBFv was unchanged. Additionally, we evaluated the cerebrovascular conductance index (CVCi = CBFv/MABP) as an indicator of the relation between CBFv and MABP after accounting for hydrostatic pressure (Immink et al. 2009). We calculated CVCi based on MABP corrected to the level of the MCA (MABP_MCA_), using the standard hydrostatic correction factor of 0.78 mmHg/cm (Van Lieshout et al. 2003) and the vertical distance from the heart (midsternum) to the TCD probe.

Transfer function analysis (TFA) across each 5‐min segment of data was performed, using the CARNet algorithm (Matlab R2016a, The MathWorks, Natick, MA) with standardized settings (Claassen et al. 2016) to calculate coherence, gain, and phase shift across the very low (0.02–0.07 Hz), low (0.07–0.20 Hz), and high (0.20–0.40 Hz) frequency ranges. Per CARNet recommendations, datasets were visually screened to exclude trials containing excessive noise and/or artifacts lasting longer than three cardiac cycles. Beat‐by‐beat data was extracted from continuous data and resampled at 5 Hz, without detrending, normalization, or filtering. Windows of 512 points were selected with 50% overlap. Triangular window smoothing and Hanning antileakage window were applied. A coherence threshold based on the 95% confidence interval was used. To evaluate the influence of posture on dynamic CA, TFA metrics across all trials were used to calculate a single average value for the supine trials and a single average value for the seated trials. Decreased phase shift along with increased gain and coherence in the very low frequency were considered indicative of less effective dynamic CA (Claassen et al. 2016).

### Statistics

Data are reported as mean ± standard deviation. Relative differences were calculated as percent change from the supine position $\left(\frac{\text{seated‐supine}}{\text{supine}}\times 100\right)$ (Serrador et al. 2000). Bivariate correlations and step‐wise multiple regression (forward and backward) were used to evaluate the influence of key variables (MABP_heart_, Q, PETCO_2_, and the hydrostatic pressure difference between the heart and MCA) on the CBFv response between supine and seated postures. Paired t‐tests were used to compare right versus left side CBFv, order effects, and metrics of CA between postures (SPSS v23, SPSS Inc., Chicago, IL). Significance was accepted at the *P* ≤ 0.05 level with Bonferroni corrections for type I error for multiple comparisons.

## Results

### Physiological responses from supine to seated positions

Unilateral CBFv measurements, ipsilateral to the finger cuff, were used for analysis, except in 3 cases where the ipsilateral signal was poor. For those cases, CBFv signals contralateral to the finger cuff were deemed suitable for substitution since no differences were observed between right and left MCAs in supine (64.6 ± 10.5 cm/s vs. 63.7 ± 12.1 cm/s, respectively; *P* = 0.99) or seated (55.9 ± 8.8 cm/s vs. 58.1 ± 10.6 cm/s, respectively; *P* = 0.08) postures. Starting position had no effect on CBFv and CA responses to changes in posture (Order Effect *P* > 0.20).

Average supine and seated values were within expected ranges for healthy individuals (Table 2). There were negligible hydrostatic differences between the heart and MCA (1.4 ± 1.4 mmHg lower at MCA) in the supine posture, thus the change in hydrostatic pressure between postures was expressed relative to a normalized value of 0 mmHg in the supine position. Relative to supine, seated measurements of CBFv (−13.0 ± 6.1%), MABP_MCA_ (−19.5 ± 8.1%), and PETCO_2_ (−5.8 ± 3.5%) were lower (*P* ≤ 0.001 for all; Table 2). No changes in Q or CVCi_MCA_ were detected (*P* > 0.05 for all; Table 2).

**Table 2:** Results of key predictor variables (PETCO_2_, MABP, hydrostatic pressure and Q) and outcome variable (CBFv). Mean ± SD, *n* = 18

	Supine	Seated	Relative differences (%)
CBFv (cm/s)	63.6 ± 10.6	55.2 ± 9.1^a^	−13.0 ± 6.1
PETCO_2_ (mmHg)	30.9 ± 2.3	29.1 ± 2.6^a^	−5.8 ± 3.5
MABP_MCA_ (mmHg)	84.2 ± 12.1	67.2 ± 7.2^a^	−19.5 ± 8.1
MABP_heart_ (mmHg)	84.2 ± 12.1	92.1 ± 8.4^a^	10.5 ± 9.9
Q (L/min)	6.6 ± 1.4	6.7 ± 1.2	4.1 ± 17.4
Hydrostatic Difference (Heart to MCA while seated, mmHg)	0	−24.9 ± 2.6^a^	N/A
CVCi_MCA_ (cm/s/mmHg)	0.77 ± 0.18	0.83 ± 0.15	9.0 ± 15.2

a *P* ≤ 0.001.

### Predictors for CBF change

**Figure 1:**
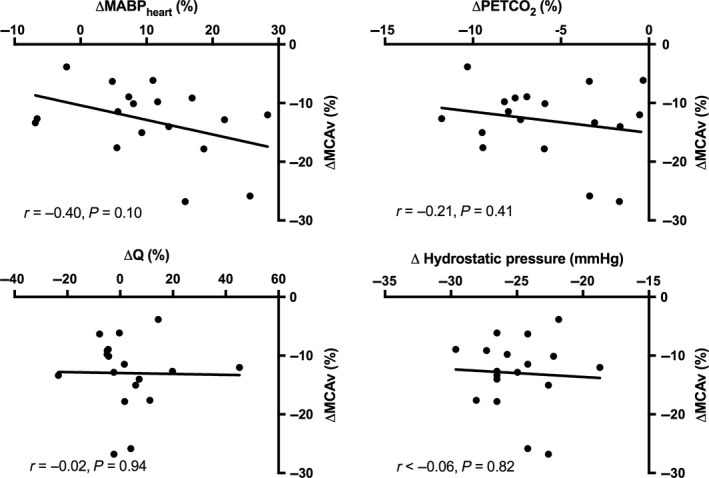
Bivariate correlations between the change in CBFv and hypothesized predictor variables (MABPheart, PETCO2, Q, and hydrostatic pressure) were weak and insignificant.

Bivariate correlations between percent changes in CBFv and four independent predictors (PETCO_2_, MABP_MCA_, Q, and hydrostatic pressure difference) were weak (range of R^2^: <0.01–0.16; all *P* > 0.05; Figure 1). Additionally, multiple linear regression revealed no combination of these variables could predict the change in CBFv (range of adjusted *R* ^2^: −0.068–0.11; all *P* > 0.05).

### Cerebral autoregulation

#### Static CA

The 19.5% decrease in MABP_MCA_ was accompanied by 13% decrease in CBFv from supine to seated; however, no difference was detected in CVCi between postures (*P* = 0.058, Table 2).

#### Dynamic CA

**Figure 2:**
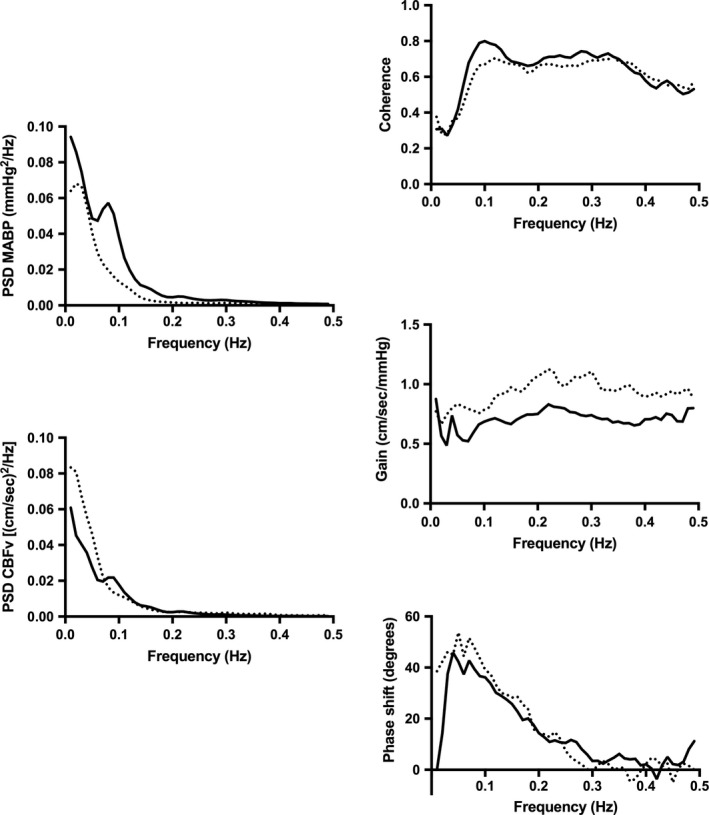
Transfer function analysis using the CARNet algorithm revealed greater power spectral density (PSD) of MABP in low and very low frequencies in the seated (solid lines) relative to supine (dashed lines) posture (*P* < 0.01). Gain across all frequencies was lower the seated relative to the supine posture (*P* < 0.01). There were no differences in PSD of cerebral blood flow velocity (CBFv), coherence or phase shift between postures.

Of the 72 possible trials (18 subjects × 2 postures × 2 repetitions), sixty‐six measurements of suitable quality for TFA processing were obtained; three supine trials and three seated trials were excluded from analysis due to excessive noise, artifact, or missing data per CARNet recommendations. Results revealed greater MABP power spectral density (PSD) in low and very low frequencies, without corresponding changes in PSD of CBFv. Transfer function metrics showed reduced gain in the MABP to CBFv spectra across all frequencies (all *P* < 0.01, Figure 2, Table 3) in the seated, relative to supine, posture. There were no differences in coherence or phase shift between postures.

**Table 3:** Transfer function analysis results using the 2016 CARNet algorithm

		Supine avg	Seated avg
MABP (mmHg^2^)		0.01 ± 0.01	0.02 ± 0.01^a^
CBFv ((cm/s)^2^)		0.01 ± 0.01	0.01 ± 0.01
PSD MABP (mmHg^2^/Hz)	0.02–0.07 Hz	0.79 ± 0.60	1.30 ± 0.69^a^
	0.07–0.20 Hz	1.00 ± 0.75	2.63 ± 1.79^a^
	0.20–0.40 Hz	0.80 ± 0.61	1.83 ± 3.51
PSD CBFv ((cm/s)^2^/Hz)	0.02–0.07 Hz	0.83 ± 0.70	0.62 ± 0.51
	0.07–0.20 Hz	1.04 ± 0.81	1.34 ± 1.17
	0.20–0.40 Hz	1.27 ± 1.33	0.71 ± 0.54
Coherence	0.02–0.07 Hz	0.45 ± 0.18	0.54 ± 0.14
	0.07–0.20 Hz	0.67 ± 0.15	0.72 ± 0.21
	0.20–0.40 Hz	0.64 ± 0.20	0.63 ± 0.20
Gain (cm/s/mmHg)	0.02–0.07 Hz	0.81 ± 0.30	0.55 ± 0.18^a^
	0.07–0.20 Hz	0.95 ± 0.31	0.72 ± 0.23^a^
	0.20–0.40 Hz	0.98 ± 0.32	0.71 ± 0.16^a^
Phase (degrees)	0.02–0.07 Hz	49.75 ± 16.24	41.74 ± 13.06
	0.07–0.20 Hz	27.87 ± 6.37	27.09 ± 9.05
	0.20–0.40 Hz	2.13 ± 5.93	5.90 ± 9.44

PSD, power spectral density. Mean ± SD, *n* = 18.

a *P* < 0.01.

## Discussion

In this study, we observed concurrent decreases in CBFv and MABP_MCA_ from a supine to seated posture, indicative of a system with imperfect static CA. Indices of dynamic CA were largely unchanged between postures. These results suggest that changes in static cerebral regulation between postures have little functional consequence for dynamic regulation of cerebral blood flow.

### Implications for static cerebral autoregulation

The observed 13% decrease in CBFv and maintenance of CVCi from supine to seated is consistent with previous research, which suggested a pressure passive relation between MABP and CBFv across the autoregulatory range (Lucas et al. 2010). Our results demonstrate a 0.76% change in CBFv per mmHg change in MABP, which is comparable to the 0.82%/mmHg change reported by Lucas et al. (2010). However, despite the similarity in average response between studies, we did not observe a correlation with changes in MABP_MCA_ and CBFv between postures (Figure 1). The absence of a strong correlation between MABP_MCA_ and CBFv does not support a pressure passive system and suggests that other variables are influencing the change in CBFv. Previous studies have tried to determine the impact of arterial carbon dioxide pressure (PaCO_2_) on CBF, estimating that it can account for up to 50% of the CBF response to postural change (Immink et al. 2006; Brothers et al. 2009; Gelinas et al. 2012). Changes in cardiac output (Q) and the hydrostatic effect have also been shown to correlate with CBF changes to varying degrees (Harms et al. 1999; Van Lieshout et al. 2001; Gisolf et al. 2004; Ogoh et al. 2015), yet to the best of our knowledge, no studies have considered the influence of more than one variable at a time. To this end, we applied step‐wise multiple linear regression to determine the relative influence of concurrent changes in MABP_MCA_, PETCO_2_, Q, and hydrostatic pressure on the change CBFv between postures. Interestingly, we were unable to identify a significant regression model (*P* > 0.05) with our data. Both forward and backward models failed to find a combination of variables that could adequately explain the variance in CBFv between postures. Qualitatively, we acknowledge that the small reduction in PETCO_2_ from the supine to seated posture likely caused mild vasoconstriction and was thus responsible for some of the reduction in CBFv. Based on an average cerebrovascular reactivity of ~2% CBFv/mmHg PETCO_2_ across the hypocapnic range, where PETCO_2_ <42 mmHg PETCO_2_ (Peebles et al. 2007), the 1.8 mmHg reduction in PETCO_2_ could have reduced CBFv by ~3.6%. This would explain ~28% of the overall reduction in CBFv between postures (3.6/13.0 = 28%). The regression model may not have been sensitive enough to detect this relationship given the large degree of individual variability in the responses. Future studies with larger sample sizes may be necessary to tease out the relative contribution of MABP, PETCO_2_, Q, and hydrostatic pressure on the change in CBFv between postures using a regression model.

### Implications for dynamic cerebral autoregulation

To investigate the impact of postural changes on dynamic CA, we used the consensus‐based CARNet TFA algorithm in an attempt to clarify some of the disparate results previously reported (Deegan et al. 2010; Romero et al. 2011; Gelinas et al. 2012). Our data show that sitting up appears to slow the frequency and increase the amplitude of oscillations in MABP, but not CBFv (Figure 2). The resulting increase in the MABP power spectrum effectively reduces the transfer function gain (i.e., how much of MABP is transferred to CBFv). Due to the change in the MABP power spectrum between postures, we do not feel it is appropriate to suggest that the reduced gain scores in the seated position reflect a meaningful change in dynamic CA, especially in light of the absence of changes in coherence and phase shift. Our results are most similar to those of Gelinas et al. (2012), who reported increased power spectral density of MABP and reduced gain in the low frequency during 90° head up tilt, relative to supine. We concur with their conclusion that these changes have little functional consequence on dynamic CA. Despite using the CARNet TFA algorithm, we echo caution in making definitive statements about dynamic CA based on TFA as the field continues to work toward a “gold standard” measurement (Claassen et al. 2016).

### Limitations

We must consider some specific limitations of this study. First, as is routinely discussed, the use of CBFv as an index for CBF must be addressed. In this study, we did not measure volumetric flow but instead measured CBFv as an index for CBF. This method relies upon the assumption of a constant vessel diameter in the MCA, which has been shown to be valid below 45 mmHg PaCO_2_ (Giller et al. 1993). Considering the widespread use of CBFv as an index for CBF and our observed values of PETCO_2_, we are confident that this limitation is not a significant hindrance to our analysis, but concede that future studies should include volumetric flow measurements. Second, noninvasive measurements may have introduced error and prevented us from detecting the influence of our key variables on CBFv. Specifically, using PETCO_2_ as an index for PaCO_2_ is well accepted in the supine posture where the two are closely matched. However, sitting up causes increased ventilation and alveolar expansion from gravity‐induced pressure gradients that lead to a ventilation‐perfusion mismatch when upright. This results in a large difference between PETCO_2_ and PaCO_2_ (Immink et al. 2006). However, when estimating PaCO_2_ using the equation from Immink et al. (2006), we still did not observe a significant correlation with CBFv (data not shown). We must also acknowledge limitations in the pulse contour analysis method used by the finger photoplethysmography device for estimating Q, compared with more accurate and invasive techniques such as thermodilution. However, Bogert et al. (2010) observed no difference between thermodilution and the pulse contour analysis method for deriving Q in either the supine or seated posture. Further, previous CA studies have used MABP as a surrogate for cerebral perfusion pressure, but changes in intracranial pressure should be considered since it is known to change with posture on the order of ~6 mmHg from supine to seated (Alperin et al. 2005).

### Summary

We observed concurrent decreases in CBFv and MABP_MCA_ from a supine to seated posture, but question whether these changes imply a pressure‐passivity of CBF in the autoregulatory range. Transfer function analysis did not demonstrate a functional change in indices of dynamic CA. Together these results suggest that changes in steady‐state cerebral hemodynamics between postures do not appear to have a large functional consequence on the dynamic regulation of CBF.

## Conflict of Interest

None.
